# Determination of urinary peptides in patients with proteinuria

**DOI:** 10.4103/0971-4065.45289

**Published:** 2008-10

**Authors:** M. Prakash, J. K. Shetty, S. Dash, B. K. Barik, A. Sarkar, R. Prabhu

**Affiliations:** Department of Biochemistry, Kasturba Medical College, Manipal, Karnataka, India; 1 Department of Nephrology, Kasturba Medical College, Manipal, Karnataka, India

**Keywords:** Urinary peptides, protein degradation, proteinuria, urinary peptides

## Abstract

Although considered useful in the diagnosis and prognosis of renal diseases, proteinuria can only be detected after significant renal paranchymal changes. There is considerable interest in the estimation of urinary peptides as an early marker of renal disease. In the current study, we have estimated urinary peptides in patients with different grades of proteinuria. Twenty-four hour urine samples were collected from 138 subjects and classified into three groups based on the urine protein excreted: group I (normoproteinuria, 0–150 mg/day, *n* = 37), group II (microproteinuria, 150–300 mg/day, *n* = 31), and group III (macroproteinuria, > 300 mg/day, *n* = 70). Urine proteins were determined using Bradford's method and urinary peptide levels were determined by subtracting Bradford's value from the Lowry value of the same sample. There was a significant decrease in the levels of urinary peptides in group III compared to group I (*P* < 0.01), however, there was no difference in peptides between groups I and II. The percentage of urinary peptides was decreased in both groups II and III compared to group I (*P* < 0.01), and there was a significant difference in % urinary peptide content in group II compared to group III (*P* < 0.01). On correlation, % urinary peptides correlated negatively with urinary proteins/g creatinine (r = - 0.782, *P* < 0.01) and positively with urinary peptides/g creatinine (r = 0.238, *P* < 0.01). Our data suggest that there is a marked decrease in urinary peptide levels with an increase in proteinuria. This may suggest impaired tubular protein reabsorption and degradation capacity of renal tubules.

## Introduction

The principal barrier to the passage of blood proteins has been thought to reside in the glomerular capillary wall. The restrictive permeability of the glomerular filter to macromolecules has been attributed to exclusion based on their size, configuration, and electrical charge.^1^ It was previously believed that most of the filtered protein reaching the renal tubule is degraded and entirely reabsorbed into the blood stream.^2^ However, recent studies suggest that 95% of albumin in humans is reabsorbed from the proximal tubules and degraded to produce small peptides (< 10 kDa) that are excreted in urine.^3^ Russo *et al*. suggested that the albumin degradation products are excreted as peptides in urine and the quantities of these peptides were in great excess of intact albumin in normal individuals.^4^ Norden *et al*. found much smaller quantities of small peptide fragments (> 250 Da) in normal urine through the use of column chromatography and mass spectroscopy.^5^

Recently, Strong *et al*. have reported increased quantities of highly degraded, tritiated albumin (150–500 Da), but no detectable intact (> 1500 Da), tritiated albumin in the urine of healthy control subjects. This was in contrast with a predominant large molecular peak of presumably undegraded, tritiated albumin with greatly diminished quantities of small fragments in a macroalbuminuric patient with diabetes.^6^ The exact anatomic location of the degradation pathway has not been determined, but it probably takes place in cells distal to the glomerular basement membrane, most probably in tubular epithelial cells, where albumin is subjected to endocytosis and trafficked to lysosomes. Once degraded, albumin degradation small peptides are subjected to exocytosis into the tubular lumen and excreted in the urine.^7^–^10^

Urinary peptides (< 10 kDa) can be detected with the use of radioactive tracers.^11^ However, urinary peptides are not detected with the use of routine total protein assays, including the sulfosalicylic acid, benzethonium, and Bradford assays, or Coomassie blue staining of electrophoretic gels. They may however, be detected with the use of assays designed to measure peptide bonds, such as the Biuret assay.^12^,^13^ Excretion rates obtained with the use of radioactive albumin and the Biuret assay have been shown to be similar in both normal control rats and those with proteinuria.^14^,^15^ Urinary peptides are also not detected with the use of conventional immunochemical assays, which can only detect intact protein or large peptides.^10^,^16^ Thus, conventional chemical and immunochemical assays severely underestimate the amount of albumin excreted in urine, and therefore, are unable to comprehensively quantify changes in urinary albumin excretion.

In the current study, we have used a modified chemical assay designed to measure urinary peptides using the Biuret principle, and also, to determine the levels of urinary peptides in normoproteinuria cases in comparison with microproteinuria and macroproteinuria cases.

## Materials and Methods

### Subjects

One hundred and thirty-eighty subjects were assigned to three groups based on their daily urine protein content. Subjects with urine protein content of 1–150 mg were categorized as group I, 150–300 mg as group II, and > 300 mg as group III. Twenty-four hour urine samples from 37 group I, 31 group II, and 70 group III cases were collected in brown bottles containing toluene as a preservative. The urine sample bottles were stored at 4°C during the period of collection. Samples were centrifuged at 3000 rpm for 10 min and analyzed immediately after the collection period. Informed consent was taken from the subjects involved in the study followed by ethical clearance from the Institutional Review Board.

### Reagents

Special chemicals such as bovine serum albumin (BSA) were obtained from Sigma^®^ Chemicals, St Louis, MO, USA. All other reagents were of analytical grade.

Protein stock: BSA was dissolved in phosphate buffered saline (PBS). Standard curves were prepared by dissolving BSA to get the following final concentrations; for Bradford assay: 2, 4, 6, 8, and 10 μg/mL; for Lowry assay: 50, 100, 150, 200, and 250 μg/mL.

For Lowry assay: we used a modified Lowry's assay for determining levels of total urinary proteins; the reagents were prepared as follows: reagent A: 2% sodium carbonate, reagent B_1_ : 1% sodium potassium tartarate, reagent B_2_ : 0.5% CuSO_4_ in reagent B_1_, reagent C: 50 mL reagent A + 1 mL reagent B_2_, and reagent D: 1N Folin-Ciocalteau reagent. Composition of Folin-Ciocalteau reagent is sodium tungstate, sodium molybdate, orthophosphoric acid, concentrated hydrochloric acid, lithium sulphate, bromine water and sodium hydroxide.

### Methods

Protein and peptide levels in urine were measured using a Genesys 10UV spectrophotometer whereas urine creatinine levels were determined by a Clinical Chemistry Automated Analyzer (Hitachi 912).

Both Lowry and Bradford assays were performed after diluting the urine samples suitably. Clear-cut dilution principles were not available in the literature to dilute urine samples for the protein and peptide assays using Lowry and Bradford's methods. Hence, we propose here a range of dilution factors based on the protein content per deciliter of the urine sample [Table 1].

**Table 1:** Dilution factors for Lowry's and Bradford's methods for diluting urine sample according to milligrams of protein per deciliter

Mg protein/dL	Dilution factor for Lowry's method	Dilution factor for Bradford's method
0–10	10	20
10–50	20	50
50–100	50	100
100–150	50	150
150–250	100	200
250–400	100	300

Urinary proteins, together with urinary peptides, were measured using the Lowry assay,^17^ whereas urinary proteins were determined using the Bradford assay.^18^ Urinary peptide levels were determined by subtracting the Bradford's value from Lowry's value of the same urine sample (Lowry value – Bradford value). All calculations were done using separate calibration curves prepared for each method.

For Lowry estimation, 0.2 mL of the diluted urine sample was taken in two sets of eppendorf tubes (sets 1 and 2) while 0.2 mL of 145 mM NaCl was taken in another tube and labeled as reagent blank (RB). To RB and to set 1, 1 mL of reagent C was added while 1 mL of reagent A was added to set 2 tubes. The tubes were shaken vigorously and incubated for 10 min at room temperature. Reagent D was added to all the tubes at the end of 10 min and the tubes were vortexed; this step is crucial for color development. The tubes were incubated at room temperature for 30 min and the absorbance was read at 600 nm.

After correcting for respective blanks, absorbance values of set 2 samples were subtracted from their counterparts in set 1. The difference in the readings arises because of the copper pretreatment of set 1 samples. The total protein contents were calculated from the calibration curves, and after multiplying with the dilution factor, the total protein content (including peptides) was expressed in gram per milliliter (g/mL) and g/g of urine creatinine.

For the Bradford assay, 1 mL of diluted urine sample was added to 1 mL of Bradford reagent. The reagent blank consisted of 1 mL of PBS and 1 mL of Bradford reagent. The contents were mixed and incubated at room temperature for 30 min and the absorbance read at 595 nm. The protein content in the sample was calculated using a calibration curve, and after multiplying with the dilution factor, values were expressed in g/mL and g/g of urine creatinine. The total urinary peptide content in the sample was calculated by subtracting the Bradford value (protein content) from the Lowry value (total protein including peptides), and the peptide content in the urine was expressed as g/mL and g/g of urine creatinine.

### Statistical analysis

Statistical analysis was done using the Statistical Package for Social Sciences (SPSS), version 10. Analysis of variance (ANOVA) followed by multiple comparison by post-hoc test was done to compare the mean values. Pearson's correlation was used to correlate the parameters. *P* < 0.05 was considered statistically significant. Microsoft Office Excel 2 was used to prepare correlation figures.

## Results

**Fig. 1:**
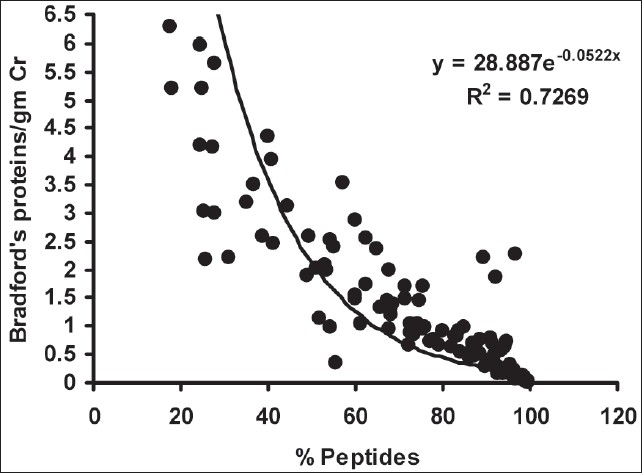
Correlation between % urinary peptides and urine protein-creatinine ratio

**Fig. 2:**
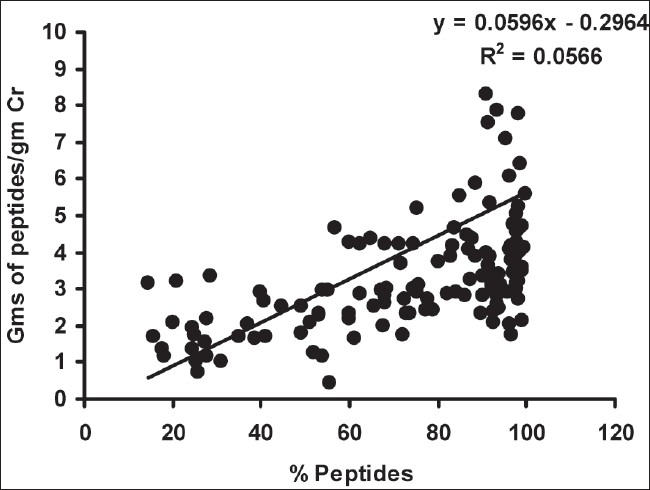
Correlation between % urinary peptides and urine peptides/g creatinine

As depicted in Table 2, there was a significant decrease in urinary peptides/g creatinine in group I compared to group III (*P* < 0.01), however, there was no such difference between groups I and II. The percentage of urinary peptides was decreased in both group II and III cases compared to group I (*P* < 0.01), and there was a significant difference in % urinary peptide content in group II compared to group III (*P* < 0.01). On correlation, the % urinary peptides correlated negatively with urinary proteins/g creatinine (r = - 0.782, *P* < 0.01) [Fig. 1] and positively with urinary peptides/g creatinine (r = 0.238, *P* < 0.01) [Fig. 2]. Urinary protein levels determined by Bradford's method were found to correlate positively with those determined by the clinical chemistry analyzer (pyrogallol red dye binding) method (r = 0.915, *P* < 0.01).

**Table 2:** Biochemical parameters in urine samples of normoproteinuria (group I), microproteinuria (group II), and macroproteinuria (group III) cases, expressed as mean ± standard error of mean (SEM), minimum and maximum values

	Normoproteinuria	Microproteinuria	Macroproteinuria
	(Group I) (n = 37)	(Group II) (n = 31)	(Group III) (n = 70)
Lowry's method (g proteins/g Cr)	6.21 ± 1.87	4.96 ± 0.76	5.75 ± 0.42
Bradford's method (g proteins/g Cr)	0.19 ± 0.06	1.05 ± 0.24	2.48 ± 0.33*
Urinary peptides (g/g Cr)	6.01 ± 1.81**	3.91 ± 0.62	3.27 ± 0.32
Bradford's method (Proteins in g/day)	0.06 ± 0.005	0.21 ± 0.008	1.70 ± 0.208
Autoanalyzer (sulfosalicylic acid) method (Proteins in g/day)	0.10 ± 0.012	0.20 ± 0.16	1.63 ± 0.206
(Lowry – Bradford) / Lowry × 100 (% urinary peptides)	97.07 ± 0.43**	83.67 ± 3.20***	58.66 ± 2.80

* *P* < 0.01 compared to microproteinuria and normoproteinuria

** *P* < 0.01 compared to microproteinuria and macroproteinuria cases

*** *P* < 0.01 compared to macroproteinuria cases

## Discussion

The determination of urinary proteins has historically been a debatable issue for many decades. Numerous methodologies have been published in the literature, proposing the added advantage of one method over another method. Most of the available methods are based on one of the following methods which include the colorimetric determination of proteins: Lowry, dye binding methods using dyes like methyl orange, bromocresol green, pyrogallol red, Biorad based on the Bradford reaction, sulfosalicylic acid, turbidimetric, or nephalometric methods.^19^ It has been recently reported that 4% or 0.6 g of the nitrogenous constituents of normal urine (undetermined nitrogen not accounted for by urea, creatinine, uric acid, or ammonia) was composed, in fact, of urinary peptides which were not determined routinely.^19^

In our study, we have determined the levels of urinary peptides as the differences of Bradford's values from Lowry's values, and we have found a significant decrease in the levels of urine peptides in macroproteinuria (Group III) cases compared to microproteinuria (Group II) and normoproteinuria (Group I) cases. A previous study by Singh *et al*.^19^ reported that, based on chromatographic and proteolytic studies, Biorad reagent used in Bradford's assay reacted only with proteins larger than 13 kDa, whereas the Lowry reagent reacted with proteins and peptides of all sizes. Based on our study report which supports the findings of Singh *et al*., it appears that most of the interference reported earlier with the Lowry and Biuret assays must have been caused by peptides in diluted urine. The assessment of lysosomal enzymes involved in peptide degradation in this setting would have provided better evidence of decreased peptide degradation, but we were not able to analyze these enzymes due to the unavailability of the method to estimate lysosomal enzymes in urine samples in our setting. The effect of diabetes mellitus on urinary peptide levels with or without renal injury also warrants further studies. We believe that our study can be taken as a pilot study in the Indian population and further studies can be designed in centers with better facilities.

Urine creatinine levels correlate positively with urine peptide levels, indicating that as the urine creatinine levels decrease due to renal injury, urine peptide levels will also be decreased due to renal paranchymal damage and the loss of protein degradative capacity of renal tubular cells. As peptide excretion may depend on the filtered load of urinary proteins (which cannot be determined directly), we have calculated peptide values as percentages of total protein material in urine (an indirect measure of the filtered load: [Lowry – Bradford]/Lowry × 100) against proteinuria. We have found significant decreases in % urinary peptides in microproteinuria and macroproteinuria cases. Determination and expression of peptides as % urinary peptides have shown significant differences among the three groups when compared to determining and expressing peptide as g peptides/g of urine creatinine. There was no difference in g of urinary peptides/g of urine creatinine between micro- and macroproteinuria cases in our study, but when the filtered load of urinary proteins was taken into consideration using the above formula, we found significant differences in % peptides that were excreted in these two groups [Table 2].

Previous authors have reported similar decreases in % urinary peptides in proteinuria cases and the decrease in % urinary peptides was found to correlate negatively with urine proteins/gm creatinine, indicating a possible relation between % urinary peptides that are excreted in urine and renal function. Significant decreases in lysosomal enzyme levels have been found in the urine of micro- and macroproteinuric patients compared to healthy controls, indicating decreased activity of tubular lysosomal enzymes.^19^ We have found that % urinary peptides correlated negatively with g of proteins/g urine creatinine, indicating a decreased presence of urinary peptides with an increase in proteinuria and the severity of renal disease. The weak positive correlation between % urinary peptides and urinary peptides/g creatinine may indicate that urinary peptides can be determined and expressed in either way. However, we believe that consideration of the filtered load of urinary proteins in determining urine peptides and expressing as % urinary peptides may well differentiate between cases in different grades of proteinuria.

We have compared clinical chemistry automated analyzer method (which use pyrogallol red dye based method to determine protein levels in urine) with Bradford's method, and have found no significant difference between the protein values obtained by these two methods; and urine protein values in both methods correlated positively. Since Bradford reagent in Bradford assay reacted only with proteins larger than 13 kDa^19^, The positive correlation between pyrogallol red based method with Bradford's method possibly indicates that the urine protein level that is determined using the pyrogallol red dye-binding method, accounts only for proteins which were greater that 13 kDa molecular weight. In support of previous reports,^13^ we speculate that most of the methods used to determine urine proteins in clinical chemistry analyzers are just estimating proteins which are larger than 13 kDa and the significant presence of peptides was underestimated by these methods. Although advanced radiological imaging techniques are available for the early detection of renal injury, they are still not available in most hospitals. Also, a serial detection of urinary peptides will be a simple, noninvasive, cost-effective method that can help in the early detection of renal parenchymal injury.

In conclusion, our study is consistent with previous studies in that there is a significant decrease in urinary peptide levels with impairment of renal function, and serial monitoring of renal function can be done by a simple determination of urine peptides. Any fall in the levels of urinary peptides from the normal range could be interpreted to indicate renal pathology at an early stage, rather than waiting for significant proteinuria. Although advanced radiological imaging techniques are available for early detection of renal injury, they are still not available in most hospitals. If sensitivity and specificity of this serial monitoring can be proved by further studies, we believe that simple serial monitoring of urinary peptides in the early detection of renal injury will be a noninvasive and cost-effective method that will be very useful in the early detection of renal injury.
